# How many hypertensive patients can be controlled in “real life”: an improvement strategy in primary care

**DOI:** 10.1186/1471-2296-14-192

**Published:** 2013-12-13

**Authors:** Alessandro Filippi, Diego Sangiorgi, Stefano Buda, Luca Degli Esposti, Giulio Nati, Italo Paolini, Antonino Di Guardo

**Affiliations:** 1 CliCon Health, Economics and Outcomes Research, Ravenna, Italy; 2 Italian College of General Practitioners, Florence, Italy

**Keywords:** Community medicine, Hypertension, Patient adherence, Practice management, Primary care

## Abstract

**Background:**

It is well known that hypertension control is non-satisfactory, but it is not clear how many hypertensive patients can be controlled in real life. We addressed this question implementing a simple, multifaceted improvement strategy in family practice.

**Methods:**

Eighteen General Practitioner (GPs) agreed upon a simple improvement strategy including: 1) the use of occasional direct/indirect contacts (prescription refilling) to decrease missing blood pressure (BP) recording, and to increase therapeutic adherence, 2) the use of home BP measurements in non-controlled patients, 3) the addition of a new drug in non-controlled, but adequately adherent patients. Results were assessed after one year by automatic data extraction from the clinical records of all hypertensive subjects.

**Results:**

The patients with a diagnosis of hypertension increased from 6.309 (age 58.5 +/- 12.4; M 45.5%) to 6.717 (age 58.6 +/- 12.9; M 45.7%): prevalence 25.3% to 27.0%. The BP recording increased: 4,305 patients (68.2%) vs 4,948 patients (78.4%) (+ 10.2%, ci 9.4%-10.9%; p < 0.001), as well as the BP control: 3,203 (50.8% of all the diagnosed hypertensive patients and 74.4% of the subjects with recorded BP value) vs 4,043 (64.1% of all the diagnosed hypertensive patients and 81.7% of the subjects with recorded BP value) (+ 13.3%, ci 12.5%-14.2%; p < 0.001 and + 7.3%, ci 6.7%-8.0%; p < 0.001).

**Conclusions:**

Almost 82% of hypertensive subjects who contact their doctors can be easily controlled. Most non-controlled patients simply don’t see their GPs; in almost all the remaining non-controlled patients GPs fail to increase drug therapy. A further improvement is therefore possible.

## Background

Hypertension is a leading modifiable risk factor for cardiovascular disease. Worldwide, 7.6 million premature deaths (about 13.5% of the global total) and 92 million DALYs (6.0% of the global total), about 54% of stroke and 47% of ischemic heart disease were attributable to high blood pressure [1]. Hypertension control is therefore a major goal for every National Health Service. In the US, improvement in hypertension detection and control has been observed in the last 30 years: the rate of hypertension increased from 23.9% in 1988-1994 to 28.5% in 1999-2000, but did not change between 1999-2000 and 2007-2008; control increased from 27.3% in 1988-1994 to 50.1% in 2007-2008 [2]. Improvement was reported for Canada: control improved from 13.2% in 1992 to 64.6% in 2009 [3]. Similar rates of control are also present in the minority of the European countries, such as Denmark, where 57% of treated patients are controlled [4]. In Italy the current control rate is 35% [5]. Despite these improvements, it is clear that hypertension treatment is still far from acceptable, although it is not known how many patients can be controlled in every-day clinical practice. This is an important piece of information, since it may help to work-out a target for quality standards and for improving strategies.

The reasons for low control rate in surveys can be divided into insufficient blood pressure measurement and recording, and insufficient treatment (prescription and adherence).

The great majority of hypertensive patients are cared for in primary care. The analysis of every-day practice shows a problem which cannot be identified by the observation of a randomly selected and carefully evaluated sample of a hypertensive population: many subjects diagnosed with high blood pressure have no up-to-date blood pressure (BP) values in their clinical records. A recent survey in Italian primary care showed that, during the last year, 16% of diagnosed hypertensive subjects didn’t visit their GPs: furthermore, of the patients who contacted their GP 16% had no recorded BP value [6]. This problem has been addressed by the British “pay for performance” strategy: in the 2009/2010 report [7] over 90% of the patients had their BP recorded in the previous 9 months. It must be noted that the reported prevalence of hypertension was 13.4%, well below the expected “real” prevalence; therefore the value and the generalizability of such a result is very difficult. Another problem is the method used to measure BP and, consequently, the cut-off value to classify a patient as controlled or not controlled. The importance of this issue is well illustrated by a Danish survey, which showed that 57% of treated patients were controlled according to office BP values, and that the percentage increased to 68% when home BP was used instead [4]. At the moment home BP monitoring? (or 24 h monitoring) isn’t recommended for all the patients with hypertension, but up to 50% of the treated population could benefit from this technique, according to the guidelines indicated: evaluation of white coat hypertension, of masked hypertension, and of resistant hypertension [8].

Other well-known obstacles to getting patients to BP target are drug under-prescription (therapeutic inertia) and poor therapeutic continuity/adherence [9]. In conclusion, it is clear that BP control in real practice cannot improve without 1) using the appropriate measurement technique, 2) knowing/recording the BP values, 3) prescribing the necessary drugs, and 4) obtaining good therapeutic continuity/adherence.

The scientific literature is rich in trials aiming to improve drug prescription, therapeutic adherence, patient involvement, and, finally, BP control. Unfortunately, a definite model is still lacking, and only general suggestions are possible, at least according to Cochrane Collaboration’s conclusion, in which he states that: “family practices and community-based clinics need to have an organized system of regular follow-up and review of their hypertensive patients…. Self-monitoring and appointment reminders may be useful adjuncts to the above strategies to improve blood pressure control, but require further evaluation” [10]. It is clear that a multifaceted strategy is needed to further improve hypertension control, and that such a strategy must be simple, cheap, and sustainable in the long run in the actual primary care working framework. A group of GPs volunteered to test such a strategy over an 18-month period, aiming to maximize BP control and to understand how many hypertensive patients can be controlled in usual clinical practice. The main results are reported in this paper.

## Methods

In Italy every resident is registered with a GP who cares for his/her patients at no charge (GPs are paid directly by the National Health Service); there is no limitation to access to office visits, and anti-hypertensive drugs are free of charge, thus eliminating two possible causes of under-treatment. Eighteen GPs, all members of the Italian College of General Practitioners (working in different towns), and particularly interested in hypertension management, decided to maximize their effort to improve BP control. They agreed upon a simple strategy, compatible with primary care organization in Italy, including the following steps: 1) appropriate technique for BP measurement, 2) systematic use of direct and indirect contacts to remind patients to regularly check BP, and to adhere to drug prescription, and 3) an increase in drug-therapy in non-controlled, adherent patients. The improvement strategy, aiming to implement better blood pressure measurement, empowerment of patients, and higher therapeutic adherence, was autonomously decided upon by the authors themselves together with the other GPs who participated in the improvement effort. This strategy was agreed upon by all the participating GPs. The treatment the patients received was consistent with good clinical practice. The clinical data were recorded according to the current standard of care, and were rendered anonymous by the GP who cared for the hypertensive patients. For these reasons, consent from patients or approval by an ethics committee was not required before the implementation of the improvement strategy, according to Italian law (law n° 196/2003; Ministry of Health Circular Letter GU n° 76/2008).

All the GPs decided to systematically identify the uncontrolled patients who needed home monitoring (or 24 hour monitoring) according to the ESH [11]. The participating GPs used two devices (Microlife Home BP ©) which automatically recorded the BP according to the recommendation of the ESH [8]; these devices were used for patients unable or unwilling to buy their own device for home measurement. All the patients received written instruction for correct BP measurement, as recommended by ESH [11]. This first intervention maximizes the use of BP measurement methods alternative to office BP measurements, which were the previous “usual care”, and contributes to an increase in patients’ empowerment. All the direct and indirect contacts (prescription re-filling without patient-doctor contact) were used to obtain missing BP values; in case of indirect contacts the GPs wrote a short note to the patient, inviting her/him to check her/his BP. All the direct and indirect contacts were also used to improve BP control: if the BP was higher than that recommended and if a reduction was clinically sound, the problem was immediately addressed (direct contact) or the patient received a short note (indirect contact), inviting her/him to see her/his GP. These contacts were also systematically used to check therapeutic adherence, verifying if the previously prescribed drugs had permitted an adequate therapeutic coverage; when necessary the therapy was reviewed with the patients, who received a written scheme of their drugs (a written scheme was also delivered in case of indirect contact for re-filling). This standardized use of indirect contacts aimed to solve a relevant problem not explicitly addressed by guidelines: reducing the number of low/non-attending patients and increasing the recorded BP values. Guidelines underscore the need for adequate adherence to therapy, but do not recommend any specific intervention. Writing a short note and adding the therapeutic scheme to the prescriptions of apparently poorly-adherent patients when re-filling prescriptions is a simple and practical way to tackle this. This is not entirely new, but the systematic use is a novelty compared with the previous “usual care”. The participating GPs agreed to increase the number/dosage of anti-hypertensive drugs after checking the correct use of the previously prescribed therapy and, of course, only when such an increase was clinically sound. This intervention simply stressed a well-known, but previously largely-ignored guideline recommendation, offering a straightforward, simple implementation strategy.

A patient was considered controlled when the office BP was < 140/90 mmHg,and/or the mean home BP was < 135/85 mmHg, and/or the mean 24 hour BP was < 126/80 mmHg [11].

The GPs began to implement this improvement strategy in June 2010; the data rendered anonymous for the final evaluation were automatically extracted from the GPs’ database on June 30^th^ 2011. The following items were extracted over the period 2009/06/01-2011/06/30: CV diseases, prescribed drugs, recorded BP and methods for measurements, as well as pertinent blood tests. Data recorded from 2009/06/01 to 2010/05/31 were used to analyse baseline practice. The last available BP (baseline and observation period) was used to classify the patient as “controlled” or “not controlled”.

Therapeutic adherence was calculated considering the single pill as the “prescribed daily dose”; only the anti-hypertensive drugs continuously prescribed from 2009/01/01 to 2011/06/30 were used to compare adherence at baseline and at the end of the observation period, and for every single patient only the drug with the best baseline adherence was considered for comparison.

### Statistics

Continuous variables were reported as mean and standard deviation, whereas categorical variables were expressed as numbers and percentages; paired t-tests and McNemar test were respectively used for comparisons. Two-tailed p-values less than 0.05 were considered significant. All analyses were performed using SPSS for Windows, version 18.0.

## Results

**Figure 1:**
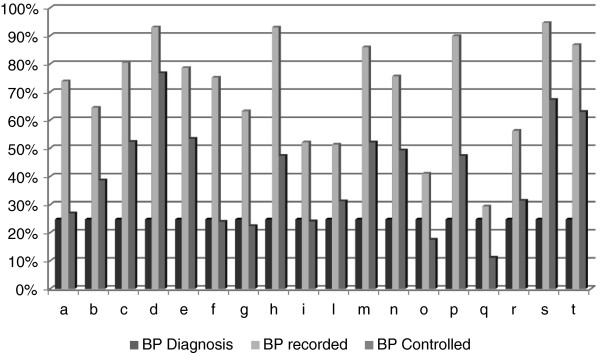
Baseline performances of the 18 participanting GPs (letters identify GPs).

The participating GPs cared for 24,918 subjects aged ≥ 15; 5 GPs worked in cities, 7 worked in towns, and 6 in small towns or semirural areas; 7 worked in Northern, 8 in Central, and 3 in Southern Italy. Their baseline performances differed widely (Figure 1), thus increasing the representativeness of the group.The number of patients with codified diagnosis of hypertension increased from 6,309 (age 58.5 +/- 12.4; M 45.5%; prevalence 25.3%) to 6,717 (age 59.6 +/- 12.9; M 45.7%; prevalence 27.0%); the main characteristics of this hypertensive population are reported in Table 1. The differences at baseline and at end of the observation period are probably due to the increased age of the population, and to the better recording of diseases for the hypertensive patients the GPs were focusing on. At baseline 4,305 patients (68.2%) had at least one recorded BP value in the previous year, and 3,203 were controlled (50.8% of all the diagnosed hypertensive patients – patients without BP records considered not controlled – and 74.4% of the subjects with at least one recorded BP value). At the end of the observation period 4,948 patients (78.4%) had at least one recorded BP value in the previous year, 4,043 were controlled (64.1% of all the diagnosed hypertensive patients – patients without BP records considered not controlled – and 81.7% of the subjects with at least one recorded BP value). The BP recording increased (+ 10.2%, ci 9.4%-10.9%; p < 0.001) as well as the BP control rate (+ 13.3%, ci 12.5%-14.2%; p < 0.001 in the whole hypertensive population – patients without BP records considered not controlled- and + 7.3%, ci 6.7%-8.0%; p < 0.001 in the patients with at least one recorded BP value). The hypertensive patients aged ≥ 80 were 1,095, and 791 (72.2%; 89.2% among the 887 patients with at least one recorded BP value) had BP < 150/90 mmHg, the target suggested by the NICE guidelines [12]. If we had considered this different cut-off for very old people there would have been a further modest increase in the total rate of controlled patients.

**Table 1:** Main characteristics of patients with recorded diagnosis of hypertension cared for by the participating GPs according to the recorded diagnoses

**Characteristic**	**Baseline (6309 patients)**	**End of the observation period (6717 patients)**
Age	58.5 (+/- 12.4);	59.6 (+/- 12.9)
Gender	Men 2871 (45,5%)	Men 3070 (45,7%)
Coronary heart disease	753 (11,9%)	934 (13,9%)
Heart failure	200 (3,2%)	252 (3,7%)
Atrial fibrillation	278 (4,4%)	376 (5,6%)
Left ventricular hypertrophy	325 (5,1%)	402 (6%)
Stroke/transitory ischaemic attacks	254 (4%)	317 (4,8%)
Renal failure	550 (8,7%)	665 (9,9%)
Diabetes mellitus	1643 (26%)	1875 (27,9%)
Chronic obstructive pulmonary disease	289 (4,6%)	363 (5,4%)

The last available BP value (which determined the “controlled” or “non-controlled” status) was ambulatory or home-based in 135 (2.1%) and 735 (11.7%) subjects. Among the patients “non-controlled” with office measurement, none had at least one previous “controlled” value with home or ambulatory monitoring.

The mean number of anti-hypertensive drug increased from 1.17 (SD 0.81; range 0-7) at baseline to 1.18 (SD 0.82; range 0-7) at the end of the observation period: + 0.01 ± 0.66 (p < 0.001); further details on drug prescription are reported in Table 2 and in Table 3.

**Table 2:** Number of prescribed anti-hypertensive compounds at baseline and at audit’s end

	**Baseline** **(mean ± SD, range)**	**Study’s end** **(mean ± SD, range)**	**p**
Total hypertensive population	1.17 ± 0.81 (0-7)	1.18 ± 0.82 (0-7)	< 0.001
Controlled	1.25 ± 0.76 (0-7)	1.27 ± 0.76 (0-5)	< 0.001
Non controlled	1.25 ± 0.87 (0-5)	1.27 ± 0.85 (0-6)	< 0.001
Without final BP recording	0.88 ± 0.86 (0-6)	0.85 ± 0.88 (0-7)	< 0.001

**Table 3:** Percentage of patients according to the number of prescribed anti-hypertensive compounds and to the patients’ final status (controlled, non- controlled, without BP recording) at baseline and at study’s end

**Baseline**	**Total**	**Controlled**	**Non controlled**	**Without recording**
0	17.3%	9.8%	15.6%	30.0%
1	56.1%	61.8%	53.9%	48.2%
2	20.7%	21.9%	23.1%	17.3%
3	5.0%	5.4%	5.8%	3.9%
4	0.9%	0.9%	1.2%	0.5%
>4	0.2%	0.2%	0.4%	0.1%
**Study’s end**	**Total**	**Controlled**	**Non controlled**	**Without recording**
0	17.2%	10.0%	13.8%	40.6%
1	55.6%	61.1%	55.6%	39.4%
2	21.0%	22.5%	22.4%	15.4%
3	5.1%	5.1%	6.5%	4.1%
4	1.0%	1.1%	1.4%	0.4%
>4	0.1%	0.1%	0.2%	0.1%

The mean number of days in therapy permitted by the recorded prescribed drugs increased from 229 to 236: + 7 ± 83 (p < 0.001); further details on adherence are reported in Tables 4 and 5.

**Table 4:** Number of days in therapy permitted by the recorded prescribed drugs (year before audit and last study year)

	**Baseline** **(mean ± SD, range)**	**Study’s end** **(mean ± SD, range)**	**p**
Total hypertensive population	229.1 ± 130.8 (0-365)	235.8 ± 128.3 (0-365)	< 0.001
Controlled	250.1 ± 116.6 (0-365)	258.6 ± 110.8 (0-365)	< 0.001
Non controlled	231.1 ± 130.0 (0-365)	241.2 ± 125.7 (0-365)	< 0.001
Without final BP record	165.7 ± 149.6 (0-365)	164.6 ± 150.8 (0-365)	< 0.001

**Table 5:** Level of adherence as percentage of day in therapy permitted by the recorded prescribed drugs (year before audit and last audit year)

	**Basal**				**Final**			
**Adherence**	**< 40%**	**40-59%**	**60-79%**	**> = 80%**	**< 40%**	**40-59%**	**60-79%**	**> = 80%**
Patients controlled*	18.4%	6.0%	20.0%	55.6%	15.4%	5.4%	22.3%	56.9%
**Patients non controlled§**	25.1%	4.4%	20.0%	50.4%	22.0%	4.8%	17.8%	55.4%
**Patients without BP record§**	45.7%	5.0%	14.0%	35.3%	46.0%	4.6%	13.7%	35.6%

*p < 0.001 § p NS.

## Discussion

The main achievement of our improvement strategy regarded home-monitoring, which was extensively used, according to the recent guidelines [9]; this is a relatively new feature in the management of hypertension in general practice, and a specific characteristic of our “new” approach to the management of hypertension. The number of patients with recorded BP values increased; this increase is statistically and clinically significant. A small improvement was also observed for therapeutic adherence; this modest, albeit statistically significant result may be due to both a greater commitment on the part of the GPs and to a greater use of home-monitoring, which, by itself, fosters awareness and the patients’ empowerment [9]. The drug prescription increase is statistically significant, but its clinical relevance is negligible; furthermore the increase is similar in both controlled and non-controlled groups. Overall, the percentage of controlled hypertensive patients increased, with both statistical and clinical significance. In conclusion: a) the method of BP measurement changed substantially, according to the guideline recommendations, b) better use of opportunistic contacts was effective in increasing the number of patients with recorded BP values, c) therapeutic adherence improved slightly, and d) drug prescription didn’t change substantially.

The improvement of BP control is mainly due to a better blood pressure measurement and only to a lesser extent perhaps to a real blood pressure reduction based on improved adherence to antihypertensive treatment.

In particular, the relevant increase in controlled patients seems to come more from the reduction of “white coat BP rise” due to appropriate BP measurement methods than from a better use of drugs, although the increased adherence may have played a role. “White coat effect on blood pressure”,’ may account for a noticeable fraction (one fourth or more) of individuals in whom hypertension is diagnosed [11]. The percentage of new controlled hypertensive patients observed in our population is perfectly compatible with the reduction of “white coat effect on blood pressure” obtained by the use of home/ambulatory BP monitoring. This isn’t a cosmetic result: detection of “white coat effect on blood pressure” avoids unnecessary, expensive and potentially dangerous overtreatment, and permits doctors to focus on patients who truly need more treatment and/or better adherence. The advantage of limiting office BP measurements has already been proven [12], and ambulatory/home BP monitoring is recommended by NICE guidelines [13] to diagnose hypertension. Our data show that this new approach to BP measurement is feasible in clinical practice and leads to a substantial advantage.

Our simple approach leads to a control rate of almost 82% in patients who contacted their GPs, and of 64% in the whole hypertensive population. These results compare favorably with those reported by a recent study in British General Practice, where patients randomized to intensive management were controlled in 63% of cases. Our achievement is even more remarkable considering that the protocol of the British study excluded the most-difficult-to-control subjects. It is probable that the use of office BP measurement as a standard to evaluate BP control can explain at least part of this difference [14].

Despite the initial agreement among the participating GPs, the increase in drug use is very small, and the association of different drugs remains sub-optimal, as well as lower than that used in the UK, according to the nhs.uk/statistics-and-data-collections [15], which reports more patients on multiple-therapy [15]. On the other hand, it must be observed that the reported hypertension prevalence in the UK is lower than ours, and it is therefore likely that this therapeutic attitude involves only part of the whole hypertensive population.

Most of our non-controlled patients use one drug, thus the most important way to further improve BP control could be to move to a two-drug therapy when one is not enough. Therapeutic inertia proves to be an obstacle that is hard to overcome, but improvement in this field could lead to an even higher BP control rate. The most useful strategy, at least in order to avoid inappropriate single-drug prescription, could be to skip the problem and to start with a solution of two-drug association when the initial value of BP shows that a single drug will not be enough to reach full control, as suggested by the European guidelines [11].

Our study presents many limits, directly related to having been conceived as a spontaneous effort to improve the personal clinical practice, and not as a formal study. For this reason no pre-specified control on the quality, and degree of practice modification was possible. Another important limit is the absence of a control group. On the other hand, comparison of relevant management changes in the same population before and after the introduction of practice modification is common in medical literature; a well-known example being the studies on the effectiveness of the “pay for performaces” contracts [16]. Despite these problems we think that the results obtained in a short period of time in a large, unselected hypertensive population implementing a very simple and non-expensive intervention can be easily generalized. Furthermore, the percentage of controlled patients could be useful when planning improving strategies and quality standards in primary care.

Finally, the problem of patients without BP recording and/or lack of contact deserve a comment. The number of patients with recorded BP increased to 78.4%. This percentage is lower than that reported by British GPs after their “pay per performances” contract, but any comparison is impaired by the very low hypertension prevalence (15,3%) according to their recorded diagnoses [17]; such a percentage may hint that a substantial number of hypertensive patients had not been included. Even if the number of patients with recorded BP increased, more than 20% of the hypertensive subjects had no useful contact with their GPs. This group has a very low therapeutic adherence, and is supposed to also have a very low control-rate. These patients represent a relevant problem both for improving BP control, and for a cost-effective use of drugs; new strategies to tackle this issue are therefore urgently needed.

## Conclusions

Extensive use of home BP monitoring and systematic use of direct and indirect contacts to improve patients’ compliance increase the BP control rate up to over 80% in unselected hypertensive subjects who contact their doctors. Despite explicit commitment on the part of the GPs to overcome therapeutic inertia, the use of multiple drugs didn’t increase, thus impairing an even higher BP control rate. Despite the GPs’ efforts, more than 20% of hypertensive patients remain unwilling/unable to contact their physicians; new strategies to tackle this problem are needed.

## Competing interests

The authors declare that they have no competing interests.

## Authors’ contributions

AF prepared the improvement strategy and wrote the paper; DS and SB performed the statistical analysis, LD contributed to writing and reviewing the paper, GN, IP, and ADG contributed to preparing the improvement strategy, supporting the participating GPs , and to reviewing the paper. All authors read and approved the final manuscript.

## Pre-publication history

The pre-publication history for this paper can be accessed here:

http://www.biomedcentral.com/1471-2296/14/192/prepub
